# Case Report: Post-operative Angioedema After a Laryngeal Mask Airway Application

**DOI:** 10.3389/fmed.2021.566100

**Published:** 2021-03-15

**Authors:** Suren Soghomonyan, Qian Fleming, Sujatha P. Bhandary

**Affiliations:** ^1^Department of Anesthesiology, Ohio State University Wexner Medical Center, Columbus, OH, United States; ^2^Department of Anesthesiology, Emory University Hospital, Atlanta, GA, United States

**Keywords:** angioedema, quincke's edema, anesthesia complications, lisinopril, laryngeal mask airway

## Abstract

Angioedema with macroglossia is a rare complication of anesthesia. We present a clinical case of post-operative development of angioedema presenting as macroglossia in a patient receiving chronic therapy with lisinopril, who developed symptoms in the early post-operative period following surgery in a lateral position, when a laryngeal mask airway was used. Possible mechanisms of angioedema and macroglossia development in our patient are discussed along with potential underlying predisposing mechanisms and available therapeutic approaches.

## Introduction

Angioedema (AE) or Quincke's edema is an acute-onset transient edema involving the skin, subcutaneous tissues, and mucous membranes of the face, oral cavity, airway structures or the gastrointestinal tract, the upper and lower extremities (1–5). The underlying mechanisms include histamine and inflammatory cytokine release, overproduction or decreased degradation of bradykinin, or a hereditary C1 esterase inhibitor deficiency: a rare disease with autosomal dominant inheritance (1, 4).

AE related to histamine release presents as an allergic reaction of an immediate type triggered by IgE-mediated release of histamine and other mediators by mast cells and basophils.

Cases of AE with increased levels of bradykinin are generally associated with use of specific medications: angiotensin converting enzyme (ACE) inhibitors, angiotensin 2 receptor antagonists, non-steroidal anti-inflammatory medications, and other drugs, including propofol, which increase bradykinin levels in tissues (1, 3, 4, 6–9). According to Ishoo E. and co-authors, ACE inhibitors are the most common cause of drug-induced AE representing 25–39% of cases (4).

Typically, propofol-induced bradykinin release is restricted to the site of injection and manifests as a transient burning sensation during drug administration. Other contributing factors include anxiety, pain, significant physical and surgical stress, infections, and temperature changes (3).

The hereditary AE due to C1 esterase inhibitor deficiency is seen in 1 in 50,000 people in the general population (10). Acquired forms of C1 esterase inhibitor deficiency have been reported and are usually associated with malignant B-cell lymphoma or several other conditions.

Literature reports describe development of AE during surgery or in the immediate post-operative period. Perioperative AE, especially if the soft tissues of face, neck, oropharynx and airway are involved, is a rare but serious complication which may require continuous monitoring and sometimes, prompt intervention to avoid devastating consequences.

While perioperative AE presenting with macroglossia has been reported in cases of general anesthesia with endotracheal intubation, there are only a few case reports when a laryngeal mask airway (LMA) was used (11–13). It is important to note that with the application of an LMA during surgery, additional mechanisms may contribute to development of macroglossia and airway compromise. An inappropriately selected size of the LMA (11) as well as patient positioning during surgery may facilitate formation of an edema and macroglossia which will be difficult to distinguish from AE affecting the tongue.

We present a clinical case of acute onset AE with macroglossia, which developed in the early post-operative period in a patient undergoing surgery in lateral position, when an LMA was used to secure the airway patency and provide ventilatory support. The patient had a history of chronic lisinopril treatment.

## Case Description

A 71 year old Caucasian male patient with a past medical history of coronary arterial disease, atrial flutter, ascending thoracic aortic dissection repair, aortic and mitral valve repair, 3-vessel coronary bypass, arterial hypertension, and bifascicular block was scheduled for an elective soft tissue biopsy of the ankle with possible resection and evacuation of the accumulated blood. He had no known history of allergies, complications of anesthesia, or any family history of angioedema. The list of home medications included: amlodipine, metoprolol, tylenol, apixaban, atorvastatin, lisinopril, spironolactone, tamsulosin, magnesium sulfate, and multivitamins. The patient weighed 90.7 kg and had a BMI of 26.39.

Following preoxygenation, anesthesia was induced with IV propofol 2 mg/kg and lidocaine 100 mg. A size 5 cuffed LMA was placed without any difficulty. The patient was then placed in right lateral position, and surgery was started. General anesthesia was maintained with inhalation of 0.8–0.9 age-adjusted minimum alveolar concentration of sevoflurane in a mixture of oxygen with air. Throughout surgery, the patient received 40 mg of ketamine in divided injections, and his blood pressure was supported with small boluses and low rate infusion of phenylephrine (0.2 mcg/kg/min). No antibiotics were administered during surgery. At the end of surgery, 8 mg of ondansetron was administered as an antiemetic. No narcotic analgesics were used, since the surgeon had used local anesthetic infiltration of the incision site during surgery.

The procedure was completed uneventfully with evacuation of collected blood and soft tissue biopsy, the LMA was removed, and the patient was transferred to the post-operative recovery unit fully awake and alert. A routine inspection of the oral cavity at the time of the LMA removal did not reveal any trauma to the mucosa or local edema.

In an hour after surgery, the anesthesiologist was notified by the recovery unit nurse that the patient complains on swelling of the tongue.

Immediate assessment revealed a progressively worsening swelling on the left half of the tongue without airway compromise. Hemodynamic and respiratory parameters remained within normal limits with SPO2 of 98%, heart rate 72/min, and normal blood pressure. In around an hour, the whole tongue became swollen, again with no signs of respiratory distress.

A preliminary diagnosis of AE was made considering the history of chronic lisinopril therapy. As alternative diagnoses, IgE-mediated anaphylactic reaction and macroglossia as a result of the LMA application and patient positioning were considered even though with lower possibility. Eight mg of dexamethasone and 25 mg of Benadryl were given IV, and inhalation of oxygen, 4 L/min was started. The otorhinolaryngology service was contacted to evaluate the patient, and emergency intubation equipment was made available. It was decided to observe the patient in the hospital setting overnight. An endoscopic examination performed by the otorhinolaryngologist revealed no signs of trauma or hematoma, the only finding was the observed isolated edema of the tongue without any airway compromise. A tryptase test was sent immediately, and the results came back normal: 4.3 mcg/L (normal values <11.0 mcg/L). The time period between the anesthesia induction and blood collection for the test was < 4 h. Empiric epinephrine was not used in our patient because of a history of significant cardiovascular disease and absence of any respiratory distress despite the lingual edema. The drug would be used as a first line choice in case of an apparent anaphylactic reaction with life-threatening airway compromise. A specific anti-bradykinin therapy with Icatibant was not used either, since the vital signs remained stable, and gradual improvement of the edema took place with time. Lastly, the drug has a relatively high cost, and its use in our patient with stable vital parameters and gradual resolution of symptoms would be questionable.

With conservative management and observation, the patient's condition improved with complete resolution of the edema within 24 h. No additional laboratory tests were ordered (measurement of C_1_ estherase inhibitor activity and the C_4_) at that time.

The patient was uneventfully discharged from the hospital next morning after an overnight observation. While in hospital, lisinopril therapy was discontinued, and the patient was referred to his primary care practitioner for further treatment and possible modifications in the medication plan.

## Discussion

AE is a symptom complex which may rarely complicate the perioperative period. In majority of cases, it remains self-limited and will only require supportive therapy with monitoring. However, in 11% of cases airway intervention is required to save the patient (1). In such patients, the mortality can be as high as 30–40% (3).

As mentioned above, three types of AE are known, and the treatment strategy will depend on etiologic mechanisms (3).

AE of allergic origin results from an allergic reaction of immediate type, when symptoms are caused by antigen-IgE mediated release of inflammatory mediators and histamine from the mast cells and basophils. In such cases, epinephrine, glucocorticoids, antihistamines (anti-H_1_ and H_2_ antagonists), and oxygen are usually effective. When indicated, bronchodilators and intravenous fluids should be administered.

Dexamethasone and Benadryl were administered to our patient, and inhalation of oxygen was initiated. Even though steroids and antihistamines are indicated only for AE of allergic origin, the possibility of acute deterioration of the respiratory function in the immediate post-operative period justified such an empiric therapy without a proof of allergic reaction. Epinephrine was not used considering adequate respiratory function during the whole period of observation and a history of significant cardiovascular disease. However, the plan was to use epinephrine should further worsening of swelling take place.

Drug-induced AE of non-allergic origin is commonly related to bradykinin overproduction or decreased metabolism. Bradykinin, increases microvascular permeability, promotes tissue edema, may induce arterial hypotension and bronchospasm. Most commonly, it is associated with the use of ACE inhibitors. The incidence of AE in patients receiving ACE inhibitors may reach 1.6%, and these drugs are believed to be the cause of drug-induced AE in 25–39% of cases (3, 4). AE in patients on such therapy may develop weeks or years after starting the treatment (4).

Other factors linked to bradykinin-mediated AE include treatment with angiotensin 2 receptor antagonists, non-steroidal anti-inflammatory drugs, latex allergy, surgical stress, and oropharyngeal instrumentation, including laryngoscopy (1). The principal therapy is discontinuation of the offending drug and oxygenation (9). Our patient had a history of chronic lisinopril therapy to control the arterial hypertension, and it is possible that lisinopril, along with surgical stress and placement of the LMA, could have contributed to development of AE.

Propofol is known to temporarily increase local bradykinin levels, which is the mechanism of pain at injection site. Even though the effect is transient, and the half-life of bradykinin is only 15 s (1), the capillary leak and edema may have a much longer duration. Propofol, in conjunction with surgical stress and mechanical manipulation during intubation, may contribute to edema formation in susceptible patients. Typically AE associated with propofol injection develops immediately following the injection (7). AE in our patient was noticed an hour following surgery, which practically excludes the possibility of AE development because of propofol injection.

Bradykinin-related AE is, in general, resistant to glucocorticoids and antihistamines. Fresh frozen plasma (FFP) has been suggested for treatment of bradykinin-induced AE, because it contains kinases accelerating bradykinin breakdown (3). However, there is a theoretical risk of AE exacerbation induced by C1 and kallikrein contained in FFP. There is some evidence that plasma pooled C1 inhibitor concentrate (C1-INH) may be effective in cases of drug-induced AE. In our case, such a treatment was not attempted, since the patient's clinical symptoms remained stable, and gradual improvement was observed.

AE of genetic origin in related to an inborn deficiency of decreased activity of C1-esterase inhibitor (1, 3, 4). These patients have a history of recurrent AE. Treatment options for hereditary AE include symptomatic management, watchful monitoring, and specific treatment with plasma pooled C1-INH (Berinert), specific kallikrein antagonist Ecallantide, and bradykinin B_2_ receptor antagonist Icatibant (3). Antifibrinolytic drugs may be used, mostly prophylactically, to block the effects of plasmin on factor XIIa and reduce bradykinin production (3). Our patient did not have any history of hereditary AE, however, familiarity with this rare disorder is important for anesthesia providers.

An important goal for an acute onset AE involving the face, neck, and oropharynx is the maintenance of airway patency and adequate respiration. All patients with AE should be monitored for airway compromise, and prompt endotracheal intubation should be performed without hesitation if the edema progresses jeopardizing respiration. When indicated, a surgical airway must be considered.

In addition to AE, there are additional mechanisms that could have contributed to the macroglossia in our patient. It is possible that lateral positioning of the patient during surgery could have caused an uneven distribution of pressures around the LMA with resultant unequal lymphatic and venous drainage from the tongue. This could possibly result in unilateral macroglossia. There are a few literature reports of perioperative macroglossia, where similar factors were mentioned as a possible explanation for perioperative macroglossia. Stillman (11) describes development of macroglossia in an infant after using an inappropriately large LMA. Patient positioning during surgery can also predispose to development of macroglossia, which will be difficult to differentiate from AE (4, 8, 11–14). In such situations, mechanical trauma related to airway manipulation and impaired venous and lymphatic drainage play a role, and, usually macroglossia is evident immediately upon completion of surgery. Removal of the endotracheal tube or LMA relieves the pressure, improves drainage, and resolves the edema within a short period of time.

The clinical presentation in our patient was different: he was asymptomatic after surgery and developed progressive swelling of the tongue shortly after it. Thus, clinical presentation of our patient along with history of lisinopril therapy favors bradykinin-mediated AE as the primary diagnosis. AE caused by an allergic reaction is another possibility, since delayed drug-induced and latex related allergies have been reported. However, the tryptase test in our patient was normal, and no hypotension, flushing, bronchospasm or other typical allergic symptoms were present. Most of allergic reactions related to anesthetics take place immediately after administration of the drug, and delayed reactions are relatively rare. A complete allergological workup would be necessary, including a repeat tryptase test done at least 24 h after the reaction, to assess the peak to background ratio and, potentially, establish the cause of AE. However, our patient was discharged next morning with a referral to his primary care practitioner, and was lost to follow-up.

This is a re-demonstration of the well-known fact that in acute care, including perioperative period, oftentimes a final diagnosis cannot be established within a short time period, and symptomatic therapy to stabilize the patient becomes a priority.

The patient required an overnight observation to prevent an acute worsening of edema and airway compromise. All the necessary equipment for immediate airway control, should the situation worsen, was readily available. Fortunately, the patient's symptoms regressed with time, and he was discharged next morning with complete resolution of edema. He was referred to his primary care practitioner for further therapy.

Icatibant, a selective, competitive B2 receptor antagonist, is recommended as an effective treatment option for bradykinin-induced AE. The drug has been approved for clinical use in the Unites States in 2019. Our patient did not receive Icatibant because of the following considerations: the patient's condition remained stable, and symptoms resolved within a day. Also, Icatibant should be used with caution in patients with cardiovascular disease (15). Lastly, the drug's high cost is a factor to consider for patients with stable clinical picture.

To our knowledge, this is the first reported case of a post-operative AE in a patient on chronic lisinopril therapy, who was operated on in lateral position with the use of an LMA. This patient case reminds of the possibility of AE in the perioperative period and highlights the importance of possible consequences. Extended monitoring, appropriate drug therapy and readiness to intervene to secure the airway if indicated is the recommended strategy. Specific therapy based on the type of AE, if known, should be used.

Our case report presents a single patient, and this is a limitation of this presentation. Because of the short duration of inpatient treatment and regression of symptoms with time, the diagnostic considerations were made on clinical judgement and limited available paraclinical data. However, AE is a rare phenomenon in perioperative setting, and even a single patient presentation will add to the current knowledge and help to better treat these patients.

This is a challenging category of patients, and focused studies are justified to develop evidence-based recommendations to manage AE in the perioperative period.

## Data Availability Statement

The raw data supporting the conclusion of this article and not covered by the patient safety and health privacy regulations will be made available by the authors, without undue reservation.

## Ethics Statement

Ethical review and approval was not required for the study on human participants in accordance with the local legislation and institutional requirements. Written informed consent for participation was not required for this study in accordance with the national legislation and the institutional requirements. Written informed consent was obtained from the individual(s) for the publication of any potentially identifiable images or data included in this article.

## Author Contributions

SS provided anesthesia, performed a literature search, and wrote the manuscript. QF participated in patient management, searched literature, and wrote the manuscript. SB provided general supervision, participated in patient management, searched literature, and wrote the manuscript. All authors contributed to the article and approved the submitted version.

## Conflict of Interest

The authors declare that the research was conducted in the absence of any commercial or financial relationships that could be construed as a potential conflict of interest.
